# High cell density and high-resolution 3D bioprinting for fabricating vascularized tissues

**DOI:** 10.1126/sciadv.ade7923

**Published:** 2023-02-22

**Authors:** Shangting You, Yi Xiang, Henry H. Hwang, David B. Berry, Wisarut Kiratitanaporn, Jiaao Guan, Emmie Yao, Min Tang, Zheng Zhong, Xinyue Ma, Daniel Wangpraseurt, Yazhi Sun, Ting-yu Lu, Shaochen Chen

**Affiliations:** ^1^ Department of NanoEngineering, University of California, San Diego, La Jolla, CA 92093, USA.; ^2^ Department of Orthopedic Surgery, University of California, San Diego, La Jolla, CA 92093, USA.; ^3^ Department of Bioengineering, University of California, San Diego, La Jolla, CA 92093, USA.; ^4^ Department of Electrical and Computer Engineering, University of California, San Diego, La Jolla, CA 92093, USA.; ^5^ School of Biological Sciences, University of California, San Diego, La Jolla, CA 92093, USA.; ^6^ Scripps Institution of Oceanography, University of California, San Diego, La Jolla, CA 92093, USA.; ^7^ Materials Science and Engineering Program, University of California, San Diego, La Jolla, CA 92093, USA.

## Abstract

Three-dimensional (3D) bioprinting techniques have emerged as the most popular methods to fabricate 3D-engineered tissues; however, there are challenges in simultaneously satisfying the requirements of high cell density (HCD), high cell viability, and fine fabrication resolution. In particular, bioprinting resolution of digital light processing–based 3D bioprinting suffers with increasing bioink cell density due to light scattering. We developed a novel approach to mitigate this scattering-induced deterioration of bioprinting resolution. The inclusion of iodixanol in the bioink enables a 10-fold reduction in light scattering and a substantial improvement in fabrication resolution for bioinks with an HCD. Fifty-micrometer fabrication resolution was achieved for a bioink with 0.1 billion per milliliter cell density. To showcase the potential application in tissue/organ 3D bioprinting, HCD thick tissues with fine vascular networks were fabricated. The tissues were viable in a perfusion culture system, with endothelialization and angiogenesis observed after 14 days of culture.

## Teaser

Living tissues with high cell density and high resolution can be 3D printed.

## INTRODUCTION

Three-dimensionally (3D) engineered tissues are artificial functional tissues composed of biomaterial scaffolds and living cells (*1*–*3*). Engineered tissues have found many biomedical applications, including basic biomedical research, disease modeling, drug testing, personalized medicine, regenerative medicine, and organ transplantation (*4*–*8*). 3D-engineered tissues are expected to accurately recapitulate the 3D architecture, cell types, and physical and biochemical environment of the native tissues, providing in vitro tissue or organ models with better biorelevance, scalability, and reproducibility compared to traditional 2D monolayer cell models or animal models (*9*). Furthermore, 3D-engineered transplantable tissues and organs developed with autologous cells may potentially mitigate the problems associated with organ donor shortage and immune rejection (*10*). Therefore, tissue engineering has attracted substantial research interest.

Native human tissues typically have a cell density on the order of 1 to 3 billion cells/ml (*11*) and have complex 3D structures with fine features on the micrometer scale. To closely recapitulate the native tissues, high cell density (HCD) is essential in many 3D-engineered tissues to establish cell-cell interactions, which are critical for the artificial tissue to mature and function. For instance, 3D-engineered cardiac tissue typically requires a cell density greater than 40 million cells/ml to enable spontaneous contraction of the tissue (*12*). In addition, using HCD tissues in drug testing could potentially improve the biochemical signal-to-noise ratio by boosting the cell population, allowing more accurate and reliable responses (*13*). In addition, HCD ensures physiological compatibility, potentially allowing functional artificial organs for implantation. Now, the typical cell density used in tissue engineering research is around 1 to 10 million cells/ml, which is two or three orders of magnitude lower than that of native tissues (*1*, *14*, *15*). Therefore, achieving HCD 3D bioprinting could potentially address various critical concerns in the state-of-the-art of tissue engineering.

Apart from cell density, fine microscale features are also critical to the native tissues’ viability and proper function. To mimic the native tissues, in tissue engineering, geometrical cues are used to guide the cells’ migration, alignment, and maturation (*4*, *16*) and are also used to guide the self-organization and morphogenesis of organoids (*17*, *18*). Furthermore, vasculature networks are essential in tissues and organs for nutrient and gas exchange (*19*–*22*). The diffusion limit for nutrient and gas exchange is between 200 and 300 μm (*22*). Traditionally, because of the lack of capability in fabricating perfusable vasculature networks in conjunction with the tissues of interest, the thickness of engineered tissues is limited by this diffusion limit (*23*, *24*). However, high-resolution 3D bioprinting enables the fabrication of vasculature networks within 3D-engineered tissues to support cell viability in the thick tissues. Hence, any improvement in biofabrication resolution can lead to transitioning away from thin tissue-engineered constructs to thick 3D-engineered tissues and even transplantable organs. Therefore, there is a need to simultaneously achieve HCD and high resolution in 3D-engineered tissues.

3D bioprinting has emerged as the most popular method to fabricate 3D-engineered tissues due to its ability to precisely deposit multiple cells and biomaterials in user-defined shapes. Various 3D bioprinting techniques have been developed in recent years, which can be broadly classified into two categories: extrusion-based printing and light-based printing (*25*, *26*). Extrusion-based bioprinting methods, including nozzle-extrusion and ink-jet methods, selectively deposit a bioink to the desired location to build a 3D construct. Because of the limitation of the physical size of the nozzle or ink-jet head, the best fabrication resolution that can be achieved is typically on the order of ~50 μm (*25*). By contrast, light-based bioprinting methods, including stereolithography, two-photon polymerization (2PP), digital light processing (DLP), and volumetric methods (*27*, *28*), selectively deliver photon energy to the desired location to locally cross-link (solidify) a bioink to fabricate a 3D structure (*29*, *30*). Since light can be precisely manipulated by optical lenses and are not limited by physical apertures, light-based bioprinting methods can achieve micrometer-scale or even submicrometer-scale nominal resolutions (*29*, *31*).

**Fig. 1.**
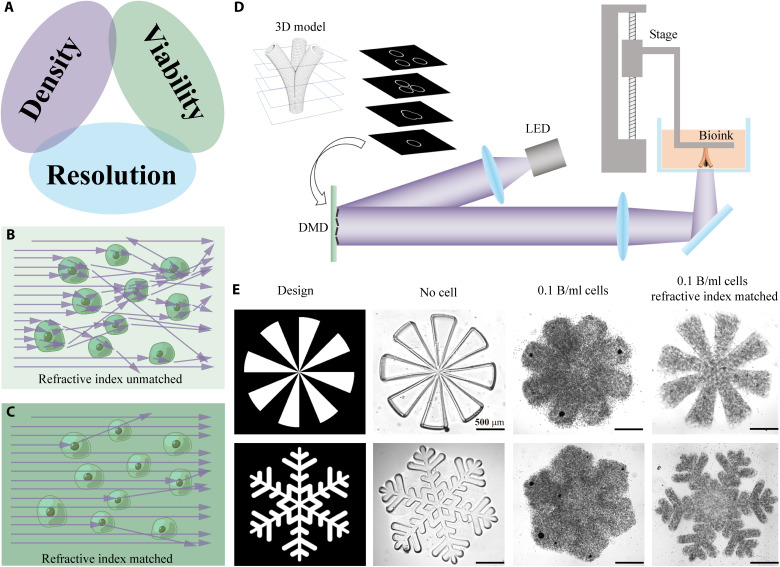
Achieving high fabrication resolution in HCD bioinks. (**A**) The “impossible trinity” in 3D bioprinting: HCD, high cell viability, and high fabrication resolution cannot be satisfied simultaneously. (**B**) A schematic showing light propagation in a refractive index–unmatched bioink. (**C**) A schematic showing light propagation in a refractive index–matched bioink, where light scattering is substanrually reduced. (**D**) A schematic showing how DLP-based 3D bioprinter works. LED, light-emitting diode; DMD, digital micromirror device. (**E**) Printing resolution comparison among three different bioink compositions: bioink without cells, bioink with 0.1 billion cells/ml, and refractive index–matched bioink with 0.1 billion cells/ml. Scale bars, 500 μm.

Although a 50-μm nominal resolution can be achieved by extrusion-based printing and micrometer-scale nominal resolution can be achieved by light-based printing, such fine features are usually achievable only under the specific conditions optimized for fabrication, where low-biocompatibility materials without encapsulated cells are used. In actual bioprinting applications where cell-encapsulated bioinks are used, the fabrication resolution often substantially deteriorates compared to the nominal situation. For extrusion-based 3D bioprinting, increasing cell density or using spheroids in the bioink requires using a bigger nozzle tip; otherwise, cell viability suffers markedly due to the shear stresses experienced during extrusion. Typically, for 10 million cells/ml or a higher density, a 200-μm or larger nozzle tip should be used, where the resulting printing resolution ranges between 200 and 500 μm (*32*, *33*). For light-based methods, because of the light scattering effect caused by the cells, the typical cell-encapsulated bioprinting resolution is a few tens to a few hundreds of micrometers (*34*–*36*). While some chemical additives, such as (2,2,6,6-Tetramethylpiperidin-1-yl)oxyl or (2,2,6,6-tetramethylpiperidin-1-yl)oxidanyl (TEMPO), can mitigate the unwanted polymerization caused by light scattering, these chemicals are usually cytotoxic (*37*, *38*). Computational approaches have also been proposed to mitigate the effect of scattering and improve the fabrication resolution of cell-laden bioinks in DLP and volumetric 3D bioprinting. However, these studies are limited to low cell density (≤10 million cell/ml) (*35*, *36*, *39*). Hence, it is difficult to fabricate a 3D-bioprinted structure that simultaneously has HCD (≥20 million cell/ml), high cell viability (≥80%), and high fabrication resolution (≤50 μm). We refer to this as the density-viability-resolution trilemma in 3D bioprinting (Fig. 1A).

DLP-based 3D bioprinting has emerged as a promising biofabrication technique due to its high resolution, high cell viability, and rapid speed (*6*). Here, we present a unique method to address the density-viability-resolution trilemma in DLP-based 3D bioprinting. By incorporating iodixanol (IDX), a biocompatible supplement to our bioink, we can precisely tune its refractive index to match that of the encapsulated cells’ cytoplasm (*40*, *41*). Bernal *et al.* (*41*) first showed that using IDX can effectively reduce the scattering of cell-laden bioink and substantially improve the fabrication quality in light-based volumetric 3D bioprinting method. However, since the traveling range of the light in the volumetric method is the entire build volume, the fabrication resolution of the volumetric method is intrinsically more severely affected by light scattering. By contrast, the traveling range of the light in the DLP method is only a layer thickness. Thus, it is less affected by light scattering. Hence, HCD, high-resolution 3D bioprinting could be easier to achieve via DLP 3D bioprinting technique.

Scattering caused by the mismatch of refractive index between the cells and their surrounding biomaterials can be minimized via refractive index tuning (Fig. 1, B and C). Measurement of the bioinks’ optical properties and simulation of light propagation confirm that IDX can effectively tune the refractive index of the bioink and, thus, substantially reducing light scattering (by ~10-fold) caused by the encapsulated cells. Here, we showcase 3D bioprinting with HCD (0.1 billion cells/ml) with a fabrication resolution of 50 μm. Immunofluorescence images and RNA sequencing (RNA-seq) also validate that healthy and functional 3D-engineered tissues can be fabricated using this approach. No statistically significant change in cells’ viability, proliferation, or phenotype was observed when incorporating IDX in the bioink. We also demonstrate that thick prevascularized tissues, with an overall size of 17 mm by 11 mm by 3.6 mm, vascular channel diameters ranging from 250 to 600 μm, with a cell density of 40 million cells/ml, can be fabricated. Endothelialization and angiogenesis were observed in these tissues after 14 days of perfusion culture.

## RESULTS

### Printing resolution

As shown in Fig. 1D, the DLP-based 3D bioprinting uses a digital micromirror device to project a 2D cross section of the 3D model to the photo-crosslinkable bioink. Upon light exposure, the photo-crosslinkable bioink, which can be either synthetic or natural, is solidified (*2*). Next, the motorized stage lifts up by one-layer thickness (typically a few tens to 200 μm) to allow uncured bioink to refill the gap. Subsequently, the next cross-sectional image is projected to the bioink, and the following layer is solidified. By repeating this process, a 3D structure can be fabricated. In the ideal condition, a newly formed layer would exactly match the shape of the projected cross section. However, in practice, incorporation of cells in the bioink causes severe light scattering, thus blurring the projected light in the bioink. Consequently, the newly formed layers cannot replicate the fine details of the projected cross-sections.

By tuning the refractive index of the bioink, the scattering effect caused by the cells in the bioink can be minimized, and the fabrication resolution can be substantially improved. Here, we demonstrate that ~50-μm feature size can be achieved in a refractive index–matched gelatin methacrylate (GelMA) bioink with a cell density as high as 0.1 billion cells/ml (see Materials and Methods for detailed bioink composition). We designed spoke-shaped and snowflake-shaped 3D structures of 250-μm thickness and 3D-printed these structures with our DLP-based 3D bioprinter using varying bioink compositions: (i) without cells, (ii) with 0.1 billion cells/ml, and (iii) refractive index–matched bioink with 0.1 billion cells/ml, respectively. Figure 1E shows the bright-field microscopic images of the printing results, where the acellular bioink has the best printing resolution, whereas the bioink with 0.1 billion cells/ml cannot resolve the spoke shape or snow shape due to light scattering. However, by tuning the refractive index of this HCD bioink, the resolution can be substantially improved, and some of the fine details of the designed structure can be resolved. Both positive features and negative features (void space) of ~50-μm size can be resolved in this manner. This demonstrates that tuning the refractive index of the bioinks can effectively improve the fabrication resolution, especially in HCD bioinks. Nonetheless, the achieved resolution by matching the index of refraction can likely be optimized depending on the cell density, material composition, structural complexity, and other key features unique to the tissue of interest.

### Optical properties

Light scattering in a cell-laden bioink originates from Rayleigh scattering and Mie scattering (*36*, *39*, *42*). Natural or modified macromolecules such as hyaluronic acid, gelatin, and collagen are commonly used in bioinks, which give rise to Rayleigh scattering. Subcellular components such as nucleus and organelles cause Mie scattering. The cytoplasm typically has a higher refractive index than the monomer solution, resulting in the situation where each cell can deflect photons passing through it akin to a microscopic lens, causing severe Mie scattering.

Cytoplasm typically has a refractive index between 1.36 and 1.39, depending on the cell types (*43*, *44*), whereas hydrogel bioinks typically have a refractive index similar to water (around 1.33). The Optiprep solution (60% IDX solution, from Sigma-Aldrich) has a refractive index of approximately 1.45 (*40*). The biopolymer concentration in the bioink only minimally affects the refractive index of the bioink. However, by adding IDX to the bioink, the refractive index of the bioink can be effectively tuned to match that of the cytoplasm (fig. S9). Therefore, scattering caused by the mismatch of refractive index between the cytoplasm and environment can be minimized (Fig. 1, B and C).

The optimal concentration of IDX in the final bioink solution depends on many factors, including the bioink composition, encapsulated cells’ type(s), osmolarity, temperature, light wavelength, etc. A pilot experiment is recommended to optimize the concentration of IDX for a given application (see Materials and Methods for recommended practice of the pilot experiment). Since the cytoplasm typically has a refractive index between 1.36 and 1.39, the optimal IDX concentration may vary from 20 to 35% (w/v).

**Fig. 2.**
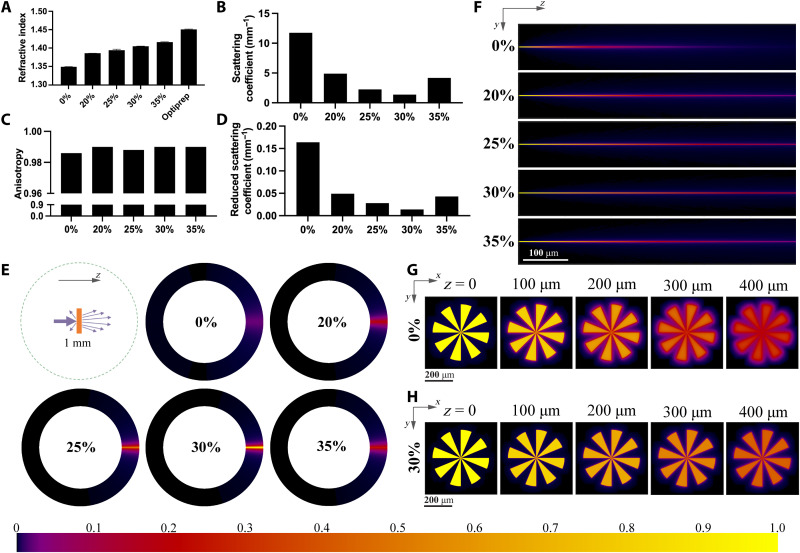
Optical properties and light energy distribution. (**A**) Refractive index of 5% GelMA bioink with various IDX concentrations at 405 nm and the refractive index of Optiprep solution (60% IDX). (**B** to **D**) Scattering coefficient, anisotropy, and reduced scattering coefficient of 40 million cells/ml cell–encapsulated bioink with various IDX concentrations. (**E**) Angular intensity distribution of the light scattered by cell-encapsulated bioink with various IDX concentrations. Light propagates in the *z* direction. (**F**) Spatial distribution (in *YZ* cross section) of light in the cell-encapsulated bioink with various IDX concentrations. (**G**) Projected pattern (in *XY* cross section) at different *z* depths of the cell-encapsulated bioink with 0% IDX. (**H**) Projected pattern (in *XY* cross section) at different *z* depths of the cell-encapsulated bioink with 30% IDX. Color bar denotes the relative light intensity in (E) to (H).

We verified that IDX can effectively tune the refractive index by measuring the refractive index of bioinks comprising 5% GelMA and various concentrations of IDX at the working wavelength of the bioprinter (405 nm). As shown in Fig. 2A, the refractive index of the bioink linearly increases when increasing the IDX concentration from 20 to 35%. The refractive indices of bioinks comprising 5% GelMA and 0% IDX, as well as the as-purchased IDX solution (Optiprep, Sigma-Aldrich) which contains 60% IDX are also plotted in Fig. 2A for reference.

We further verified that a proper concentration of IDX can effectively reduce the bioinks’ scattering effect. The scattering effect of the material is usually characterized by scattering coefficient, anisotropy, and reduced scattering coefficient. We prepared bioinks comprising 5% GelMA, 40 million cells/ml, and various IDX concentrations and used an approach combining Monte Carlo simulation and particle swarm optimization algorithm to determine the bioink’s scattering properties at the working wavelength of the bioprinter (405 nm). The measured scattering coefficient, anisotropy, and reduced scattering coefficient of these bioinks are shown in Fig. 2 (B to D). The high scattering coefficients and the anisotropy values that are close to 1 suggest that the cell-laden bioinks are highly light scattering, with mostly forward scattering. The bioink without refractive index tuning has a scattering coefficient of 11.76 mm^−1^ and a reduced scattering coefficient of 0.164 mm^−1^. By tuning the refractive index with 30% IDX, the scattering coefficient of the bioink substantially decreases to 1.377 mm^−1^, and the reduced scattering coefficient decreases to 0.014 mm^−1^, which means that this approach can reduce scattering by approximately 10-fold (also see visual appearance comparison in fig. S1). By carefully tuning the concentration of IDX, scattering can potentially be further reduced.

To provide a more intuitive result to visualize how the refractive index–matched bioink can reduce the scattering effect, we also used a Monte Carlo approach to simulate the angular distribution and spatial distribution of the scattered light. Figure 2E and fig. S6 are the simulated angular distribution of travel directions of those photons’ passing through a 1-mm-thick bioinks containing 40 million cells/ml and various IDX concentrations, and Fig. 2F is the simulated spatial distribution of the photons inside the bioink. We can see that the photons widely spread out when traveling in the 0% IDX bioink while they are still highly aligned in the 30% IDX bioink. Furthermore, we designed a spoke-like pattern to be projected onto the bioink and assumed that this pattern is infinitely collimated. The simulation results (Fig. 2, G and H) show that this pattern quickly blurred out in the bioink with 0% IDX, while it can mostly resolve its details in the bioink with 30% IDX.

### Biocompatibility

IDX has been generally considered biocompatible and nontoxic—it is commonly used as a contrast agent to facilitate angiography, sold under the trade name Visipaque, as well as a density gradient medium under the trade name Optiprep (*45*). It is iso-osmolar and chemically inert, making it compatible with a wide range of bioinks and cell types.

**Fig. 3.**
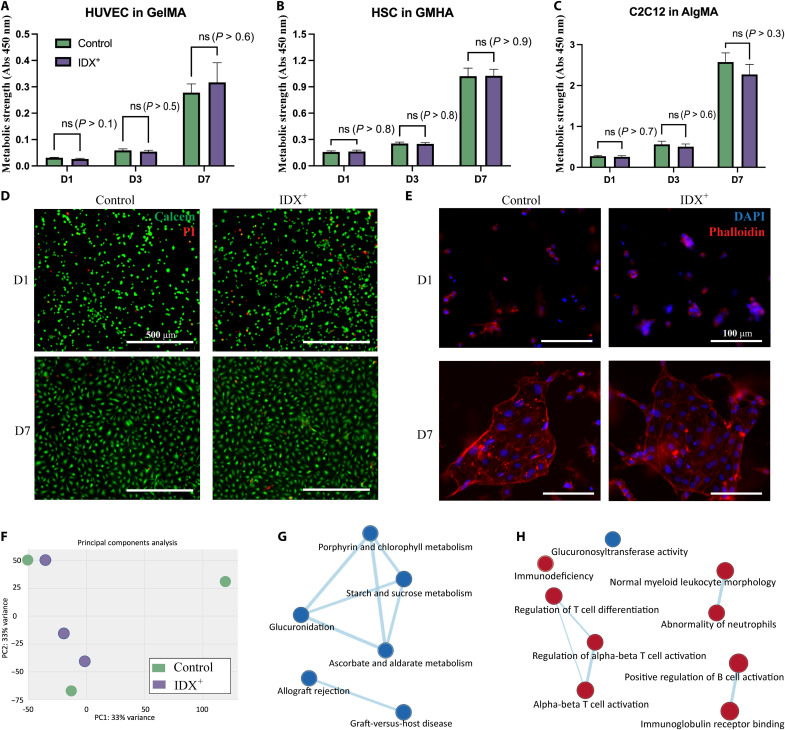
Biocompatibility analysis. (**A** to **C**) Comparison of metabolic strength of the printed tissues using bioinks with or without IDX. (**D**) Live (green) and dead (red) cells of the printed tissues using bioinks with or without IDX. (**E**) Comparison of the images of cytoskeleton of the printed tissues using bioinks with or without IDX. (**F**) Principal components analysis results of HUVEC slabs using bioinks with or without IDX (*n* = 3). (**G**) Network analysis of enriched gene sets in the Molecular Signature Database curated collection. (**H**) Network analysis of enriched gene sets in the ontology collection. Red nodes, up-regulated gene sets; blue nodes, down-regulated gene sets. Abs, absorbance. ns, not significant.

To verify IDX’s biocompatibility with various biomaterials and cell types, we performed bioprinting of thin slabs using three commonly used formulas: human umbilical vein endothelial cells (HUVECs) in GelMA, human Schwann cells (HSCs) in glycidyl methacrylate hyaluronic acid (GMHA), and C2C12 in alginate methacrylate (AlgMA). Since the ideal IDX concentration range varies between 20 and 35%, we chose to compare 3D-printed tissues in which 0 or 35% IDX was used (see Materials and Methods for detailed bioink composition). Metabolic activity strength measured using the Cell Counting Kit-8 (CCK-8) assay reveals exponential growth of the encapsulated cells in all three types of bioinks during the 7 days of culturing. Furthermore, using IDX has no statistically significant effect (*n* = 6, *P* > 0.1 for all pairs) on cells’ metabolic activity compared to controls, meaning that IDX did not hinder the proliferation of cells (Fig. 3, A to C).

In addition, live/dead staining was also used to characterize cell viability. HUVEC in GelMA bioink is printed as thin slabs with either 0 or 35% IDX. Overall, the majority of the cells are alive for both 0 and 35% IDX samples on days 1 and 7 of culture (Fig. 3D). No significant qualitative difference was observed between 0 and 35% IDX, meaning that incorporation of IDX was found to not substantially affect cell viability. Furthermore, the cytoskeleton of HUVECs was stained using phalloidin to visualize the cells’ morphology. HUVECs in both experimental conditions demonstrated endothelialization on day 7, with no qualitative differences observed (Fig. 3E).

In addition to confirming that HUVEC viability and angiogenesis were not notably inhibited in IDX-incorporated bioinks versus that of our controls, we also evaluated whether any phenotypic and metabolic alterations were induced by the presence of IDX. This is especially important for vasculature-on-a-chip studies, where the metabolism and immune-regulation of the endothelium is of interest. Clinically observed adverse effects and toxicological studies have identified elevation of oxidative stress as the main mechanism of IDX-induced endothelial dysfunction, and heme oxygenase-1 is up-regulated to counteract the injury (*46*–*49*). Here, RNA-seq was performed to comprehensively investigate the potential changes caused by IDX exposure in the HUVECs in 3D-printed GelMA hydrogel.

Differential expression was first investigated with DESEQ2. In the principal components analysis, the samples did not cluster into two groups (Fig. 3F), indicating that substantial phenotype alternation was not induced by IDX. On the other hand, 587 genes have been significantly up-regulated and 569 genes down-regulated, with a cutoff of |log_2_ – fold change| > 0.5849 and *P* < 0.05 (data S1). To further evaluate the biological impact that IDX brought to the bioprinted vasculature, gene set enrichment analysis (GSEA) using the Molecular Signature Database was used. In the hallmark gene set collection [h.all.v7.4.symbols.gmt (Hallmarks)], xenobiotic metabolism, heme metabolism, and complement gene sets were identified as significantly enriched in the samples printed with bioink containing IDX with false discovery rate < 25% and *P* < 0.05 (fig. S2). The up-regulation of xenobiotic metabolism indicated that biotransformation and the relevant enzyme expression were activated by IDX. The heme metabolism was a result of the oxidative stress (*50*) induced by the IDX, and the enrichment in the complement system is potentially related to inflammatory responses. These findings correlate well with the clinically observed adverse effects and previous toxicological studies of IDX (*46*–*49*). Since the enrichment was observed in samples collected 7 days after bioprinting when IDX should have been fully dissipated from the slabs, it is necessary to take the delayed and long-lasting molecular changes in HUVECs into consideration for further studies.

Within the curated gene set collection [c2.all.v7.4.symbols.gmt (Curated)], 3 gene sets were identified to be up-regulated, and 11 gene sets were down-regulated in response to IDX exposure (data S2); in the ontology gene set collection [c5.all.v7.4.symbols.gmt (gene ontology)], 18 gene sets were identified to be up-regulated, and 1 gene set was down-regulated (data S3). As highlighted by the network analysis by Cytoscape, porphyrin and chlorophyll metabolism, glucuronidation, starch and sucrose metabolism, and ascorbate and aldarate metabolism were down-regulated (Fig. 3G), along with the down-regulation of glucuronosyltransferase activity (Fig. 3H), which is potentially related to the activation of xenobiotic metabolism. Meanwhile, the allograft reject and graft-versus-host disease gene sets were down-regulated (Fig. 3G), while a group gene sets related to acquired immunity (regulation of T cell differentiation, alpha-beta T cell activation, and regulation of alpha-beta T cell activation; normal myeloid leukocyte morphology and abnormality of neutrophils; and immunoglobulin receptor binding and positive regulation of B cell activation) was up-regulated, accompanied by the up-regulation of immunodeficiency (Fig. 3H), implying an immunosuppressive regulatory role of the endothelium induced by IDX.

Collectively, several notable changes in metabolic and immune-regulatory activities of the endothelial cells were observed upon exposure to IDX during bioprinting, which should be taken into consideration in further studies in which pharmacokinetics and pharmacodynamic (PK/PD), immunotherapy evaluation, and pathological progression are of the focus. However, the incorporation of IDX in the bioink did not hinder the viability and endothelialization of the HUVECs, and the phenotype of the HUVECs showed minimal change due to exposure to IDX.

### Vascularized thick tissue printing

Thick tissues refer to those engineered tissues with their sizes beyond the diffusion limit (200 to 300 μm). Because of inefficient nutrient and gas exchange, necrosis is inevitable in a solid thick tissue. Therefore, vasculature networks are essential in thick engineered tissues or organs. Although 3D-printed porous structures such as 3D lattices or log pile geometries can well support the cell viability in thick engineered tissues under an in vitro culture condition, the absence of vascular networks makes them unable to be integrated with the host vasculature upon transplantation (*24*, *51*). Thus, biofabrication of prevascularized thick tissue has attracted intense research interest. To date, direct 3D fabrication of vasculature networks with HCD remains a major challenge. Some studies use sacrificial material to cast the vasculature networks, followed by dissolving the sacrificial templates (*14*, *19*, *52*, *53*). Since the sacrificial materials contain no or low density of cells, a high fabrication resolution can be achieved. However, the postfabrication processes involving sacrificial template removal and endothelial cells seeding/settling are complicated and time consuming (a few hours to a day), and the perfusion culture cannot be started until finishing these postprocessing steps. As a result, the cell viability within the thick tissues is compromised. In addition, because of the effect of gravity, the seeded endothelial cells will be nonuniformly deposited in the vascular network (*54*). Hence, direct 3D printing of prevascularized tissues with encapsulated endothelial cells provides a more promising approach, because the encapsulated endothelial cells can migrate to and proliferate at the lumen, enabling endothelialization of the prevascularized network and promoting angiogenesis (*55*).

**Fig. 4.**
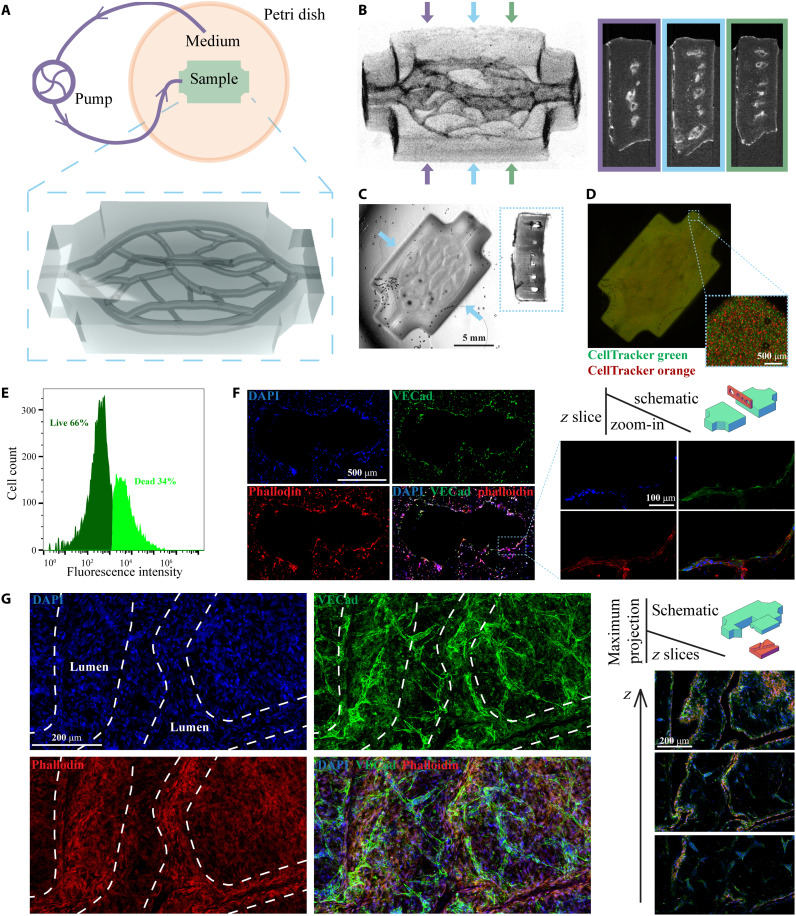
3D printing of vascularized perfusable thick tissues. (**A**) Schematic of the perfusion culture system and the 3D render of the printed tissue. (**B**) μCT images of the printed samples (perspective view and cross sections). (**C**) Bright-field images of the printed samples (top view and cross section). (**D**) Fluorescence images of the printed samples. (**E**) Cell viability in the thick tissue after 14 days of perfusion culture. (**F**) Immunofluorescence images of a cryosection in a plane perpendicular to the printed channels. (**G**) Immunofluorescence images of a horizontally cleaved chunk. The *z* slices are in horizontal planes, and the maximum projection images are stacks of all *z* slices. The white dashed lines denote the position of the 3D-printed lumen.

Because of the density-viability-resolution trilemma caused by cell-induced light scattering, previous research on direct 3D printing of prevascularized tissues using light-based methods is usually limited to either no/low cell density or poor fabrication resolution (*22*, *34*, *56*, *57*). We designed and 3D-printed a thick (17 mm by 11 mm by 3.6 mm) prevascularized tissue construct (Fig. 4A) using our refractive index–matched bioink containing 40 million cells/ml. The diameters of the hollow vascular channels range from 250 to 600 μm. HUVECs and human dermal fibroblasts (HDFs) were encapsulated in the GelMA bioink at 23 and 17 million/ml density, respectively (see Materials and Methods for detailed bioink composition). Figure 4B shows the micro–computed tomography (μCT) images (perspective view and cross sections) of the 3D-printed structure, and Fig. 4C shows the bright-field microscopic images (top view and cross section) of the printed structure. Hollow channels were observed in the scaffold, supporting the claim that desired complex microstructural features can be printed in cellularized scaffolds with high resolution and high fidelity. Figure 4D is the fluorescence image of the printed tissue, where the two cell types were stained with CellTracker Green and CellTracker Orange, respectively. This confirms that high-density, uniformly mixed cells were encapsulated in the printed structures.

Because of the vasculature networks, prevascularized 3D tissues have a much larger surface area than nonvascularized 3D tissues; thus, the gas and nutrient exchange efficiencies can be greatly improved. Although the diffusion limit can be overcome by adding vasculature networks to the 3D-engineered tissues, under static culture conditions, the exchange efficiencies are still insufficient to support the viability of HCD thick tissues. Therefore, it is necessary to use perfusion culture with the prevascularized tissues. Gravity-driven perfusion is widely used for perfusion culture. However, for thick tissues with complex vasculature networks, the pressure provided by gravity is insufficient to drive the culture medium through such high-resistance vasculature networks (*54*, *58*, *59*). A perfusion culture driven by a peristaltic pump is an alternative solution that provides increased control over the system and scalability (*54*). We used a microfluidic peristaltic pump to actively pump the culture medium through the vasculature network immediately after 3D bioprinting (Fig. 4A). The system was maintained for 14 days in an incubator, and then the printed construct was harvested. Flow cytometry was used to count the number of live/dead cells (stained with a Zombie Green viability kit) in the harvested tissue. A 66% of live cells (Fig. 4E) indicates that cells were highly viable in this thick tissue across the 14-day perfusion culture, suggesting that necrosis was avoided because of the 3D-printed vasculature and perfusion culture.

The harvested samples went through immunofluorescence staining to evaluate changes in cell morphology and function in response to perfusion culture. DAPI (4′,6-diamidino-2-phenylindole) and phalloidin were used to stain the nucleus and cytoskeleton, and VE-cadherin was used to label the cellular junctions between HUVECs. Immunofluorescence images of cryosection in a plane perpendicular to the channels are shown in Fig. 4F, at which two vascular channels are merging/splitting. A monolayer of HUVECs was observed around the channel’s cross section, confirming the endothelialization of the 3D-printed vascular channels. In addition, immunofluorescence images from a horizontally cleaved chunk are shown in Fig. 4G and fig. S8. Images from various horizontal planes (*z* slices) further confirm that a dense and uniform monolayer of endothelial cells was formed along the printed lumen. Angiogenesis beyond the printed lumen was observed in the maximum projection images stacking all *z* layers, indicating potential sites of spontaneous formation of new capillaries. These immunofluorescence images confirm that the cells in the 3D-printed vascular networks are viable, functional, and replicate key morphological changes that are observed in vascular networks in vivo.

## DISCUSSION

3D bioprinting techniques that can achieve HCD and high resolution simultaneously are highly demanded in tissue engineering. In extrusion-based 3D bioprinting, when printing with an HCD, large nozzles need to be used to preserve cell viability, leading to poor fabrication resolution. In light-based 3D bioprinting, light scattering caused by the embedded cells also substantially decreases the resolution. Hence, HCD, high cell viability, and high fabrication resolution cannot be pursued simultaneously in current 3D bioprinting techniques.

We demonstrated that by tuning the refractive index of the bioinks through the addition of IDX in DLP-based 3D bioprinting, HCD and better fabrication resolution can be achieved. We demonstrated that the light scattering effect was substantially reduced (by ~10-fold), and the fabrication resolution can be substantially improved. Tuning the refractive index of bioink with IDX is a widely applicable method, which can be applied to most cell types and hydrogel bioinks. The refractive indices of most hydrogel bioinks are lower than that of the cytoplasma, and the biopolymer concentration only minimally affects the refractive index (fig. S9). IDX can effectively increase these bioinks’ refractive indices to match that of the cytoplasm. However, note that the bioinks’ scattering mainly comes from two aspects: refractive index mismatch between the cytoplasm and the surrounding biomaterial and subcellular components such as nucleus and organelles. This approach can effectively reduce the scattering from the former aspect, yet it has no effect on the scattering from the latter aspect. Thus, in a bioink with a very HCD (much greater than 0.1 billion cells/ml), although the total scattering can be effectively reduced, the remaining scattering could still substantially affect the printing quality.

The addition of IDX slightly increases the bioink’s viscosity (fig. S10A). If a high-viscosity bioink is desired, other additives could be considered to be added. For instance, xanthan gum and glycerol were used to improve the homogeneous distribution of cells and reduce cell clumping (*34*). While the DLP 3D bioprinting resolution could be substantially improved on the *XY* plane, the *z* (the direction of light propagation) resolution mainly depends on the material’s light attenuation properties. Stronger light attenuation leads to a shorter light penetration depth and thus finer *z* resolution. Light-absorbing dyes have been widely used to increase the light absorption of the bioinks and thus improve the *z* resolution (*34*). However, this also results in a longer printing time as the layer thickness is decreased. Since a prolonged printing time (>20 min) could compromise the cell viability, it is important to keep a good balance between the *z* resolution and the printing time.

We also demonstrated that the refractive index tuning agent IDX is biocompatible, with negligible effect on cell viability, proliferation, and phenotype. We also identified the potential alternation of metabolic and immune-regulatory function caused by IDX exposure, which should be taken into consideration in further studies where PK/PD, immunotherapy evaluation, and pathological progression are involved. On the other hand, since the HUVEC transcriptome response to IDX is comparable to the reported IDX toxicology and side effects, the IDX primed vasculature is potentially helpful in the prediction of treatment response for the diseases that usually involved angiography for diagnosis.

We further demonstrated bioprinting of an HCD, prevascularized thick tissue using this approach. HUVECs and HDFs were encapsulated in the bioink. Such tissues were viable in a perfusion culture system, and endothelialization and angiogenesis of HUVEC were observed after 14 days of culture without the need to seed cells. The printed tissues maintained their structural integrity well after 14 days of perfusion culture, and the printed vasculature remained perfusable after harvest.

This approach could be applied to other light-based 3D printing techniques besides the DLP method, including stereolithography, 2PP, and volumetric method. The efficacy of this approach in volumetric 3D bioprinting has been demonstrated (*41*). While the volumetric method has a faster fabrication speed than DLP, its fabrication resolution is intrinsically more vulnerable to light scattering than the DLP method due to its long light traveling range in the bioink. That means, for the same bioink with a certain degree of light scattering, the DLP method could reach a finer resolution than the volumetric method. Or, under the same requirement of the fabrication resolution, the DLP method could use a bioink with a higher cell density than that of the volumetric method. The application of IDX in stereolithography or 2PP methods has not been studied yet. However, since these methods use a raster scanning style fabrication, their fabrication speed is much slower than the DLP or volumetric method, which could limit their applications in 3D bioprinting even if their fabrication resolution could be improved by adding IDX.

There are a few limitations associated with this approach. Since this approach is based on the DLP 3D printing technique, its bioink utilization rate could be lower than that of the extrusion-based 3D bioprinting. However, it has a much higher resolution and much faster fabrication speed than extrusion-based methods. In addition, when printing large-scale, highly overhanging soft structures, poor mechanical strength could lead to deformation or failure during printing. Light-based volumetric 3D printing methods could potentially address this issue. However, fabrication resolution of the volumetric printing method is more severely affected by light scattering due to its long light penetration depth. Overall, this work represents the state-of-the-art technique achieving both high resolution and HCD in 3D bioprinting.

We have achieved 50-μm resolution in an IDX-added GelMA bioink with the cell density of 0.1 billion cells/ml. In addition, we were able to 3D print GelMA cylinder structures (1.5 mm in diameter and 1 mm in height) with a cell density as high as 0.225 billion cells/ml without compromising the structural integrity (fig. S10, C and D). These HCD-engineered tissues are soft (their Young’s moduli are around 1 kPa) (fig. S10B) but stable, and they did not disassemble during manipulation. Although such soft constructs are difficult to manipulate when free standing, they are easy to manipulate if printed on coverslips. While their cell densities are still below what is observed physiologically (1 to 3 billion cells/ml), this technique enables 3D bioprinting with HCD, high viability, and high resolution simultaneously. This technique is straightforward and generalizable and can be easily applied to most biomaterials and cell types. It is an important step toward being able to fabricate functional large scale, clinically transplantable tissues or organs, where HCD and fine vascular networks are essential.

## MATERIALS AND METHODS

### Bioink composition

Typically, bioinks for light-based 3D bioprinting consist of monomers/oligomers, photoinitiators, solvents, cells, and other additives. In this study, we used various monomers/oligomers, including GelMA, GMHA, and AlgMA. We used lithium phenyl-2,4,6-trimethylbenzoylphosphinate (LAP) as a photoinitiator and phosphate-buffered saline (PBS) as a solvent. We used various cell types, including 293Ts, HUVECs, HDFs, C2C12, and HSCs. We used IDX as a refractive index tuning agent. Detailed bioink composition for each experiment is elaborated below.

The acellular bioink used in resolution test (Fig. 1E) consists of 5% (w/v) GelMA, 0.6% (w/v) LAP, and PBS as a solvent. The cell-laden bioink in this experiment consists of 5% GelMA, 0.6% LAP, 293T cells (0.1 billion cells/ml), and PBS as the solvent. The cell-laden refractive index–matched bioink in this experiment consists of 5% GelMA, 0.6% LAP, 293T cells (0.1 billion cells/ml), 30% IDX, and PBS as the solvent.

The bioink used in the refractive index measurement (Fig. 2A) consists of 5% GelMA, 0, 20, 25, 30, or 35% IDX, and PBS as the solvent. The bioink used in the scattering coefficient measurement (Fig. 2, B to D) consists of 5% GelMA, 0, 20, 25, 30, or 35% IDX, 293T cells (40 million cells/ml), and PBS as the solvent.

The bioink used in the HUVEC viability experiment (Fig. 3 A, D, and E) consists of 5% GelMA, 0.6% LAP, HUVEC cells (2 million cells/ml), 0 or 35% IDX, and PBS as the solvent. The bioink used in the HSC viability experiment (Fig. 3B) consists of 2.5% GMHA, 0.6% LAP, HSC cells (10 million cells/ml), and 0 or 35% IDX; in addition, 0.5% GelMA is also added to the solution to improve cell adhesion. The bioink used in the C2C12 viability experiment (Fig. 3C) consists of 1% AlgMA, 0.6% LAP, C2C12 cells (10 million cells/ml), and 0 or 35% IDX; in addition, 0.25% GelMA is also added to the solution to improve cell adhesion. The bioink used in the HUVEC RNA-seq experiment (Fig. 3F) consists of 5% GelMA, 0.6% LAP, HUVEC cells (10 million cells/ml), 0 or 35% IDX, and PBS as the solvent.

The bioink used in the thick tissue printing experiment (Fig. 4) consists of 5% GelMA, 0.6% LAP, 1% yellow food dye, 30% IDX, HUVEC (23 million cells/ml), HDF (17 million cells/ml), and PBS as the solvent. The bioink used in the rheology testing (fig. S10A) consists of 5% GelMA and 0 or 30% IDX. The bioink used in the Young’s modulus testing (fig. S10B) consists of 5% GelMA, 0.6% LAP, 30% IDX, and 293T cells (0 cell/ml, 40 million cells/ml, 0.1 billion cells/ml, or 0.225 billion cells/ml).

GelMA, GMHA, and LAP were synthesized following published protocols (*60*–*62*). The IDX solution was purchased from Sigma-Aldrich (Optiprep, D1556-250ML, Sigma-Aldrich). AlgMA was a gift from Allegro 3D Inc. (San Diego, CA).

### Determining the optimal concentration of IDX

Cytoplasm typically has a refractive index between 1.36 and 1.39. Assuming that IDX linearly changes the refractive index of the bioink, the concentration of IDX should be in the range between 20 and 35%. However, the optimal concentration is difficult to determine before 3D bioprinting since it depends on many factors including the bioink composition, cell type, osmolarity, temperature, light wavelength, etc.

A good way to determine the optimal concentration is to make bioinks with a series of IDX concentrations and without photoinitiator and then use an optical goniometer type setup to measure the scattered light distribution of a 1-mm-thick bioink. Another less accurate method is to fire laser through a 1-cm cuvette containing the bioink and then use a camera that can lock the exposure value to record the scattered light spot (fig. S3). We do not recommend using total transmittance or total reflectance to determine the optimal IDX concentration, because their change is quite small when using different IDX concentrations.

### Refractive index measurement

The refractive index of bioink is measured using a homemade refractometer with a 405-nm laser in three technical replicates. As shown in fig. S4A, the laser beam is focused on the hypotenuse of a right-angle prism, which is made of N-SF11 glass and has a refractive index of 1.8421 at 405 nm. The converging beam enters the prism-sample interface at different angles, and total internal reflection (TIR) occurs at some of these angles. The diverging beam exits the prism, is collimated by a lens, and collected by a camera. The pattern collected by the camera shows the boundary where TIR happens (fig. S4B), and the critical angle of TIR can be determined on the basis of this pattern (fig. S4C). To accurately relate the TIR boundary position on the captured image to the actual reflecting angle, water and isopropanol, whose refractive index and thus TIR critical angles are known, were used to calibrate this relation. The refractive index of water, isopropanol, and N-SF11 glass can be found at https://refractiveindex.info.

### Scattering measurement and simulation

In general, it is challenging to directly measure the scattering coefficient and anisotropy in a highly scattering specimen such as a biological tissue or HCD bioink. Indirect methods involving numerical simulation are usually required to accurately analyze these properties (*42*, *63*).

We used a well-developed 3D Monte Carlo method (*64*, *65*) combined with a particle swarm optimization algorithm (*66*) to numerically calculate the scattering properties of the cell-laden bioinks. The total transmittance and total reflectance at 405 nm of the 1-mm-thick bioink containing 40 million cells/ml were measured using a Ultraviolet-Visible-Near infrared (UV-Vis-NIR) spectroscope (PerkinElmer, Lambda 1050) with integrating sphere (the mean of three replicates was used). The angular intensity distribution of the scattered light at 405 nm was measured with a homemade optical goniometer type setup (the mean of three replicates was used) (fig. S5). Given arbitrary optical properties, the total transmittance, total reflectance, and angular distribution can be calculated by the Monte Carlo method. We then used a particle swarm optimization algorithm on MATLAB (MathWorks, Natick, MA) to acquire the optical properties that best fit the measured total transmittance, total reflectance, and angular distribution. Figure S6 plots the same dataset as in Fig. 2E in line graphs instead of color maps and shows that the simulated angular distribution of the scattered light well fits the measurement values. Next, we used the acquired scattering coefficient and anisotropy to simulate the spatial distribution of light inside the bioink with Monte Carlo method (Fig. 2F). This spatial distribution is also the point spread function of scattering. Hence, the pattern scattering results (Fig. 2, G and H) are the convolution of this point spread function and the original pattern.

### Cell culture

Primary HUVECs and primary HDFs were purchased from Cell Application Inc. and cultured per the manufacturer’s protocol. Briefly, HUVECs were maintained in the endothelial growth medium (211-500, Cell Application Inc.), fed with fresh medium every 2 days, and passaged 1:5 every 4 days. HDFs were maintained in the fibroblast growth medium (116-500, Cell Application Inc.) and passaged 1:3 every 3 days. Cells under passage 7 were used for bioprinting and further studies.

HSC, C2C12, and 293T were purchased from American Type Culture Collection and cultured in Dulbecco’s modified Eagle medium (11995-065, Gibco) supplemented with 10% (v/v) fetal bovine serum (10438026, Gibco). The cells were passaged every 3 days.

Before printing, the cells were washed with Dulbecco’s PBS (DPBS; 14190144, Gibco) and disassociated with 0.25% trypsin-EDTA (25200072, Gibco) for 3 min in a 37°C CO_2_ incubator, followed by neutralization with their culture media and resuspension in the bioink. 3D-printed thin slabs were rinsed with PBS three times to remove the IDX residue, then cultured in an incubator, and fed with fresh medium every 2 days.

### Tissue perfusion culture

3D-printed thick tissues were briefly rinsed and then immediately connected to a perfusion culture system using a peristaltic pump (*54*, *67*) to continuously feed fresh medium through the vascular channels at a flow rate of 0.2 ml/min (Fig. 4A and fig. S7A). Thirty milliliters of endothelial growth medium was added into the reservoir (petri dish). Medium was changed three times in the first hour of perfusion culture to remove the residual IDX and was changed every 2 days during the subsequence culture period. The tissues were harvested at day 14.

The perfusion culture system was constructed as following: Using an fused deposition modeling (FDM) 3D printer (Prusa MK3S+, Prusa Research), an open-top fluidic manifold was constructed out of polyethylene terephthalate glycol (fig. S7D). The square hole in the center accommodates the glass coverslip substrate upon which the DLP 3D-printed tissue scaffold is adhered to. The open channels radiating away from the center provide multiple degrees of freedom through which perfusion tubing can be threaded to interface with the scaffold’s inlet/outlet. The manifold’s total diameter is small enough to be placed inside a standard 90-mm petri dish, in which 30 ml of media was placed as a reservoir.

Using an stereolithography (SLA) 3D printer (Form 3+, Formlabs) and open-source protocols (*67*), we constructed a small-scale multichannel microfluidic peristaltic pump (fig. S7C). The central component of the system is composed of an NEMA 17 stepper motor. The motor is connected to a 3D-printed central shaft mounted on bearings to allow free spinning; the central shaft itself has six stainless steel rollers, also mounted on bearings to allow free spinning. This assembly is then enclosed within a grooved manifold through which peristaltic tubing can be threaded. Programmable control of the motor’s operation then rotates the central shaft and associated rollers against the peristaltic tubing, resulting in the repeated roller-induced contraction/expansion cycles on the tubing that produce a peristaltic flow.

Immediately after DLP 3D bioprinting, the IDX-modified, HUVEC-laden tissue scaffold adhered to the glass coverslip was placed within the open-top fluidic manifold. Peristaltic tubing [silicone, inside diameter (ID), 1 mm; McMaster-Carr] threaded through the pump was connected to the tissue scaffold’s inlet via Tygon tubing (ID, 0.5 mm; Cole-Parmer) and secured using tissue adhesive (3M VetBond) (fig. S7B).

### RNA sequencing

HUVECs were 3D-printed in 250-μm slabs using GelMA bioink with 0 or 35% IDX. After 7 days of culture, the RNAs from the 3D-printed slabs are extracted with TRIzol reagent (15596018, Ambion) followed by purification using a spin-column method with Direct-zol RNA Microprep (R2060, Zymo Research). The RNA quality evaluation and sequencing were performed by Novogen Inc. with three biological replicates of each condition. The sequencing data were analyzed with FastQC, trimmed with Trimmomatic, aligned with HISAT2, and annotated with StringTie. Differential gene expression was analyzed with DESeq2. GSEA was performed with GSEA_4.2.3 (Broad Institute). Network analysis was performed with Cytoscape 3.9.1 using the EnrichmentMap application.

### Viability assay

Cell viability in the printed thin slabs was quantified with CCK-8 (K1018, ApexBio) with six biological replicates of each condition. At designated time points, the slabs are washed with DPBS, incubated with 1 ml of fresh media with 10% CCK-8 reagent at their regular incubation condition for 45 min. After incubation, 200 μl of the supernatant was collected from each sample, and their absorbance at 450 nm was measured with Tecan Infinite 200 PRO.

The cell viability was also evaluated with Live/Dead staining. At designated time points, the slabs were washed with DPBS twice and incubated with 2 μM calcein AM (C3099, Invitrogen) and 3 μM propidium iodide (P3566, Invitrogen) in the fresh culture media at their regular incubation condition for 30 min. After incubation, the slabs were washed with DPBS twice and imaged with a fluorescence microscope (Leica DMI6000B).

Cell viability in the thick tissue was characterized by a Zombie Green viability kit (423111, BioLegend). The tissue was cultured in a perfusion culture system for 14 days and then harvested. A thin section was taken from the middle portion of the sample using a scalpel. The section was stained with a Zombie Green viability kit, next fixed with 4% paraformaldehyde (PFA), and then digested using 0.25% trypsin-EDTA to remove the GelMA. Cells were strained using a 40-μm filter before loading onto the BD Accuri C6 Plus Flow Cytometer. Fifteen thousand cells were collected for analysis. Cells were first gated to exclude the cell debris. The remaining cells were gated on the basis of the fluorescent intensity in the FL1 channel (488 nm).

### μCT imaging

The soft hydrogel samples need to be scanned in an aqueous environment; otherwise, they substantially deform. However, the radiology contrast between the GelMA hydrogel and water is very low. Therefore, a non–water-soluble contrast agent is needed. In this study, we chose BaCO_3_ as the contrast agent. The sample was first soaked in 1% (w/v) BaCl_2_ solution and then transferred to 1% (w/v) Na_2_CO_3_ solution, immediately followed by Na_2_CO_3_ perfusion. Thus, the external surface of the construct and the internal surface of the vascular network were coated with BaCO_3_. Samples were scanned using a Skyscan 1076 μCT scanner (Bruker, Konich, Belgium) immersed in PBS in a custom-designed 3D-printed container. Samples were scanned at 9 μm by 9 μm by 9 μm voxel size, applying an electrical potential of 50 kVp, a current of 200 μA, 180° in 0.8° steps, and using a 0.5-mm Al filter. All μCT image processing was performed using MATLAB. Volumetric data were reconstructed and viewed using the Volume Viewer application.

### Immunofluorescence staining and imaging

The thick tissues were processed into cryosections or chunks followed by immunofluorescence staining and imaging.

#### Cryosection

The harvested samples were fixed with PFA for 30 min and then soaked overnight in 30% sucrose solution at 4°C on a nutating tube rocker. Next, they were immersed in optimal cutting temperature compound (23-730-571, Fisher Scientific) and placed in a cryostat set at −20°C. Cryosections of 40- or 60-μm thick were made and placed on poly-l-lysine (0.1%, w/v)–coated slides.

The cryosectioned samples were gently washed with DPBS, permeabilized with 0.1% Triton X-100 (T8787, Sigma-Aldrich), and blocked with 2% bovine serum albumin (A2153, Sigma-Aldrich). Primary rabbit VE-cadherin antibody (2158, Cell Signaling Technology) was diluted 1:200 in cell staining buffer (420201, BioLegend) and incubated with the samples at 4°C overnight. The primary antibody was then labeled with donkey anti-rabbit immunoglobulin G CF543 secondary antibody (20308-1, Biotium), which was diluted in cell staining buffer at 1:200 and incubated at 37°C for 2 hours. Cytoskeleton and nuclei were labeled with Phalloidin eFluor 660 (50-6559-05, eBioscience) and DAPI (4083S, Cell Signaling Technology) per the manufacturer’s instruction before the slides were mounted with antifade reagent (9071S, Cell Signaling Technology).

#### Chunk

The harvested samples were fixed with PFA for 30 min and vertically cut into four pieces using a scalpel. Next, the small pieces were horizontally split into two halves to obtain chunks that expose half of the printed vascular channels. These chunks were cleared using a tissue clearing kit (CytoVista, V11322, Thermo Fisher Scientific) to facilitate imaging per the manufacturer’s protocol. The primary and secondary antibody were diluted 1:200 in the antibody dilution buffer, respectively. Phalloidin 660 and DAPI were diluted 1:1000 and coincubated with the secondary antibody.

In the cryosection process, the samples slightly swelled (~20%) due to the sucrose treatment. In the chunk process, the sample substantially shrank (~2× to 3×) due to tissue clearing. Therefore, Fig. 4 (F and G) does not represent the actual size of the as-printed samples. Figure 4C represents the actual size of the as-printed samples. The stained samples were imaged on a Leica SP8 fluorescence confocal microscope, a Leica DMI6000B fluorescence microscope, and a Keyence BZX800 fluorescence microscope.

### Mechanical property testing

Young’s moduli of the printed structures were measured with a MicroSquisher by CellScale using the ramp function in displacement mode per the manufacturer’s manual. DLP-printed cylinders with a diameter of 1.5 mm and a height of 1 mm were printed for testing. The compression magnitude was set at 200 μm; both loading and recovery were 25 s (0.125 s/μm). The test was performed with triplicate samples of each condition and repeated in three technical replicates.

The rheological properties of GelMA bioinks were measured by a rotational rheometer (Discovery Hybrid Rheometer HR 30, TA Instruments), equipped with a cone geometry with a diameter of 40 mm and an angle of 2°, and a Peltier plate with temperature control. The gap between the cone geometry tip and the Petier plate was 52 μm for all measurements. The viscosity was measured by varying the shear rate from 1 to 1000 s^−1^ with the flow ramp mode with three technical replicates.

### Statistical analysis

Refractive indices (Fig. 2A and fig. S9), Young’s moduli (fig. S10B), and viscosity (fig. S10A) were presented as means ± SD. Cell viability tests (Fig. 3, A to C) were presented as means ± SE, where six biological replicates were used, and pairwise comparison with Student’s *t* test (two tailed) was performed to evaluate the statistical significance. *P* values < 0.05 were considered as significant.

## References

[1] J. M. Unagolla, A. C. Jayasuriya, Hydrogel-based 3D bioprinting: A comprehensive review on cell-laden hydrogels, bioink formulations, and future perspectives. Appl. Mater. Today 18, 100479 (2020). 32775607 10.1016/j.apmt.2019.100479 PMC7414424

[2] C. Yu, J. Schimelman, P. Wang, K. L. Miller, X. Ma, S. You, J. Guan, B. Sun, W. Zhu, S. Chen, Photopolymerizable biomaterials and light-based 3D printing strategies for biomedical applications. Chem. Rev. 120, 10695–10743 (2020). 32323975 10.1021/acs.chemrev.9b00810 PMC7572843

[3] S. V. Murphy, A. Atala, 3D bioprinting of tissues and organs. Nat. Biotechnol. 32, 773–785 (2014). 25093879 10.1038/nbt.2958

[4] W. Zhu, K. R. Tringale, S. A. Woller, S. You, S. Johnson, H. Shen, J. Schimelman, M. Whitney, J. Steinauer, W. Xu, T. L. Yaksh, Q. T. Nguyen, S. Chen, Rapid continuous 3D printing of customizable peripheral nerve guidance conduits. Mater. Today 21, 951–959 (2018). 10.1016/j.mattod.2018.04.001 PMC6538503 31156331

[5] Z. Zhong, J. Wang, J. Tian, X. Deng, A. Balayan, Y. Sun, Y. Xiang, J. Guan, J. Schimelman, H. Hwang, S. You, X. Wu, C. Ma, X. Shi, E. Yao, S. X. Deng, S. Chen, Rapid 3D bioprinting of a multicellular model recapitulating pterygium microenvironment. Biomaterials 282, 121391 (2022). 35101743 10.1016/j.biomaterials.2022.121391 PMC10162446

[6] H. H. Hwang, S. You, X. Ma, L. Kwe, G. Victorine, N. Lawrence, X. Wan, H. Shen, W. Zhu, S. Chen, High throughput direct 3D bioprinting in multiwell plates. Biofabrication 13, 025007 (2021). 10.1088/1758-5090/ab89ca 32299077

[7] M. E. Kupfer, W.-H. Lin, V. Ravikumar, K. Qiu, L. Wang, L. Gao, D. B. Bhuiyan, M. Lenz, J. Ai, R. R. Mahutga, D. Townsend, J. Zhang, M. C. McAlpine, E. G. Tolkacheva, B. M. Ogle, In situ expansion, differentiation, and electromechanical coupling of human cardiac muscle in a 3D bioprinted, chambered organoid. Circ. Res. 127, 207–224 (2020). 32228120 10.1161/CIRCRESAHA.119.316155 PMC8210857

[8] N. Noor, A. Shapira, R. Edri, I. Gal, L. Wertheim, T. Dvir, 3D printing of personalized thick and perfusable cardiac patches and hearts. Adv. Sci. 6, 1900344 (2019). 10.1002/advs.201900344 PMC6548966 31179230

[9] E.-T. Verjans, J. Doijen, W. Luyten, B. Landuyt, L. Schoofs, Three-dimensional cell culture models for anticancer drug screening: Worth the effort? J. Cell. Physiol. 233, 2993–3003 (2018). 28618001 10.1002/jcp.26052

[10] A. Parihar, V. Pandita, A. Kumar, D. S. Parihar, N. Puranik, T. Bajpai, R. Khan, 3D printing: Advancement in biogenerative engineering to combat shortage of organs and bioapplicable materials. Regen. Eng. Transl. Med. 8, 173–199 (2022). 34230892 10.1007/s40883-021-00219-w PMC8252697

[11] J. D. Enderle, J. D. Bronzino, *Introduction to Biomedical Engineering* (Elsevier/Academic Press, ed. 3, 2012).

[12] K. L. Miller, Y. Xiang, C. Yu, J. Pustelnik, J. Wu, X. Ma, T. Matsui, K. Imahashi, S. Chen, Rapid 3D bioprinting of a human iPSC-derived cardiac micro-tissue for high-throughput drug testing. Organs-on-a-Chip 3, 100007 (2021).

[13] R. Cheong, A. Rhee, C. J. Wang, I. Nemenman, A. Levchenko, Information transduction capacity of noisy biochemical signaling networks. Science 334, 354–358 (2011). 21921160 10.1126/science.1204553 PMC3895446

[14] M. A. Skylar-Scott, S. G. M. Uzel, L. L. Nam, J. H. Ahrens, R. L. Truby, S. Damaraju, J. A. Lewis, Biomanufacturing of organ-specific tissues with high cellular density and embedded vascular channels. Sci. Adv. 5, eaaw2459 (2019). 31523707 10.1126/sciadv.aaw2459 PMC6731072

[15] Z. Zhang, Y. Jin, J. Yin, C. Xu, R. Xiong, K. Christensen, B. R. Ringeisen, D. B. Chrisey, Y. Huang, Evaluation of bioink printability for bioprinting applications. Appl. Phys. Rev. 5, 041304 (2018).

[16] X. Ma, S. Dewan, J. Liu, M. Tang, K. L. Miller, C. Yu, N. Lawrence, A. D. McCulloch, S. Chen, 3D printed micro-scale force gauge arrays to improve human cardiac tissue maturation and enable high throughput drug testing. Acta Biomater. 95, 319–327 (2019). 30576862 10.1016/j.actbio.2018.12.026 PMC6584548

[17] T. Takebe, J. M. Wells, Organoids by design. Science 364, 956–959 (2019). 31171692 10.1126/science.aaw7567 PMC8212787

[18] M. Nikolaev, O. Mitrofanova, N. Broguiere, S. Geraldo, D. Dutta, Y. Tabata, B. Elci, N. Brandenberg, I. Kolotuev, N. Gjorevski, H. Clevers, M. P. Lutolf, Homeostatic mini-intestines through scaffold-guided organoid morphogenesis. Nature 585, 574–578 (2020). 32939089 10.1038/s41586-020-2724-8

[19] J. S. Miller, K. R. Stevens, M. T. Yang, B. M. Baker, D.-H. T. Nguyen, D. M. Cohen, E. Toro, A. A. Chen, P. A. Galie, X. Yu, R. Chaturvedi, S. N. Bhatia, C. S. Chen, Rapid casting of patterned vascular networks for perfusable engineered three-dimensional tissues. Nat. Mater. 11, 768–774 (2012). 22751181 10.1038/nmat3357 PMC3586565

[20] J. D. Baranski, R. R. Chaturvedi, K. R. Stevens, J. Eyckmans, B. Carvalho, R. D. Solorzano, M. T. Yang, J. S. Miller, S. N. Bhatia, C. S. Chen, Geometric control of vascular networks to enhance engineered tissue integration and function. Proc. Natl. Acad. Sci. U.S.A. 110, 7586–7591 (2013). 23610423 10.1073/pnas.1217796110 PMC3651499

[21] D. B. Kolesky, R. L. Truby, A. S. Gladman, T. A. Busbee, K. A. Homan, J. A. Lewis, 3D bioprinting of vascularized, heterogeneous cell-laden tissue constructs. Adv. Mater. 26, 3124–3130 (2014). 24550124 10.1002/adma.201305506

[22] W. Zhu, X. Qu, J. Zhu, X. Ma, S. Patel, J. Liu, P. Wang, C. S. E. Lai, M. Gou, Y. Xu, K. Zhang, S. Chen, Direct 3D bioprinting of prevascularized tissue constructs with complex microarchitecture. Biomaterials 124, 106–115 (2017). 28192772 10.1016/j.biomaterials.2017.01.042 PMC5330288

[23] T. Takebe, K. Sekine, M. Enomura, H. Koike, M. Kimura, T. Ogaeri, R.-R. Zhang, Y. Ueno, Y.-W. Zheng, N. Koike, S. Aoyama, Y. Adachi, H. Taniguchi, Vascularized and functional human liver from an iPSC-derived organ bud transplant. Nature 499, 481–484 (2013). 23823721 10.1038/nature12271

[24] N. F. Huang, V. Serpooshan, V. B. Morris, N. Sayed, G. Pardon, O. J. Abilez, K. H. Nakayama, B. L. Pruitt, S. M. Wu, Y. Yoon, J. Zhang, J. C. Wu, Big bottlenecks in cardiovascular tissue engineering. Commun. Biol. 1, 199 (2018). 30480100 10.1038/s42003-018-0202-8 PMC6249300

[25] W. Zhu, X. Ma, M. Gou, D. Mei, K. Zhang, S. Chen, 3D printing of functional biomaterials for tissue engineering. Curr. Opin. Biotechnol. 40, 103–112 (2016). 27043763 10.1016/j.copbio.2016.03.014

[26] H. H. Hwang, W. Zhu, G. Victorine, N. Lawrence, S. Chen, 3D-printing of functional biomedical microdevices via light- and extrusion-based approaches. Small Methods 2, 1700277 (2018). 30090851 10.1002/smtd.201700277 PMC6078427

[27] P. N. Bernal, P. Delrot, D. Loterie, Y. Li, J. Malda, C. Moser, R. Levato, Volumetric bioprinting of complex living-tissue constructs within seconds. Adv. Mater. 31, 1904209 (2019). 10.1002/adma.201904209 31423698

[28] B. E. Kelly, I. Bhattacharya, H. Heidari, M. Shusteff, C. M. Spadaccini, H. K. Taylor, Volumetric additive manufacturing via tomographic reconstruction. Science 363, 1075–1079 (2019). 30705152 10.1126/science.aau7114

[29] S. You, J. Li, W. Zhu, C. Yu, D. Mei, S. Chen, Nanoscale 3D printing of hydrogels for cellular tissue engineering. J. Mater. Chem. B 6, 2187–2197 (2018). 30319779 10.1039/C8TB00301G PMC6178227

[30] S. You, K. Miller, S. Chen, Chapter 1. Microstereolithography, in *Biomaterials Science Series*, D.-W. Cho, Ed. (Royal Society of Chemistry, 2019); 10.1039/9781788012683-00001, pp. 1–21.

[31] S. You, W. Zhu, P. Wang, S. Chen, Projection printing of ultrathin structures with nanoscale thickness control. ACS Appl. Mater. Interfaces 11, 16059–16064 (2019). 30964636 10.1021/acsami.9b02728

[32] D. Murata, K. Arai, K. Nakayama, Scaffold-free bio-3D printing using spheroids as “Bio-Inks” for tissue (re-)construction and drug response tests. Adv. Healthc. Mater. 9, 1901831 (2020). 10.1002/adhm.201901831 32378363

[33] C. Mandrycky, Z. Wang, K. Kim, D.-H. Kim, 3D bioprinting for engineering complex tissues. Biotechnol. Adv. 34, 422–434 (2016). 26724184 10.1016/j.biotechadv.2015.12.011 PMC4879088

[34] B. Grigoryan, S. J. Paulsen, D. C. Corbett, D. W. Sazer, C. L. Fortin, A. J. Zaita, P. T. Greenfield, N. J. Calafat, J. P. Gounley, A. H. Ta, F. Johansson, A. Randles, J. E. Rosenkrantz, J. D. Louis-Rosenberg, P. A. Galie, K. R. Stevens, J. S. Miller, Multivascular networks and functional intravascular topologies within biocompatible hydrogels. Science 364, 458–464 (2019). 31048486 10.1126/science.aav9750 PMC7769170

[35] J. Madrid-Wolff, A. Boniface, D. Loterie, P. Delrot, C. Moser, Controlling light in scattering materials for volumetric additive manufacturing. Adv. Sci. 9, 2105144 (2022). 10.1002/advs.202105144 PMC9353445 35585671

[36] J. Guan, S. You, Y. Xiang, J. Schimelman, J. Alido, X. Ma, M. Tang, S. Chen, Compensating the cell-induced light scattering effect in light-based bioprinting using deep learning. Biofabrication 14, 015011 (2022). 10.1088/1758-5090/ac3b92 PMC8695056 34798629

[37] S. Suri, L.-H. Han, W. Zhang, A. Singh, S. Chen, C. E. Schmidt, Solid freeform fabrication of designer scaffolds of hyaluronic acid for nerve tissue engineering. Biomed. Microdevices 13, 983–993 (2011). 21773726 10.1007/s10544-011-9568-9 PMC8638827

[38] J. T. Toombs, M. Luitz, C. C. Cook, S. Jenne, C. C. Li, B. E. Rapp, F. Kotz-Helmer, H. K. Taylor, Volumetric additive manufacturing of silica glass with microscale computed axial lithography. Science 376, 308–312 (2022). 35420940 10.1126/science.abm6459

[39] S. You, J. Guan, J. Alido, H. H. Hwang, R. Yu, L. Kwe, H. Su, S. Chen, Mitigating scattering effects in light-based three-dimensional printing using machine learning. J. Manuf. Sci. Eng. 142, 081002 (2020).

[40] T. Boothe, L. Hilbert, M. Heide, L. Berninger, W. B. Huttner, V. Zaburdaev, N. L. Vastenhouw, E. W. Myers, D. N. Drechsel, J. C. Rink, A tunable refractive index matching medium for live imaging cells, tissues and model organisms. eLife 6, e27240 (2017). 28708059 10.7554/eLife.27240 PMC5582871

[41] P. N. Bernal, M. Bouwmeester, J. Madrid-Wolff, M. Falandt, S. Florczak, N. G. Rodriguez, Y. Li, G. Größbacher, R. Samsom, M. van Wolferen, L. J. W. van der Laan, P. Delrot, D. Loterie, J. Malda, C. Moser, B. Spee, R. Levato, Volumetric bioprinting of organoids and optically tuned hydrogels to build liver-like metabolic biofactories. Adv. Mater. 34, 2110054 (2022). 10.1002/adma.202110054 35166410

[42] S. You, P. Wang, J. Schimelman, H. H. Hwang, S. Chen, High-fidelity 3D printing using flashing photopolymerization. Addit. Manufacturing 30, 100834 (2019). 10.1016/j.addma.2019.100834 PMC7442265 32832382

[43] W. Choi, C. Fang-Yen, K. Badizadegan, S. Oh, N. Lue, R. R. Dasari, M. S. Feld, Tomographic phase microscopy. Nat. Methods 4, 717–719 (2007). 17694065 10.1038/nmeth1078

[44] M. Schürmann, J. Scholze, P. Müller, J. Guck, C. J. Chan, Cell nuclei have lower refractive index and mass density than cytoplasm. J. Biophotonics 9, 1068–1076 (2016). 27010098 10.1002/jbio.201500273

[45] M. Reed, P. Meier, U. U. Tamhane, K. B. Welch, M. Moscucci, H. S. Gurm, The relative renal safety of iodixanol compared with low-osmolar contrast media. JACC Cardiovasc. Interv. 2, 645–654 (2009). 19628188 10.1016/j.jcin.2009.05.002

[46] Y. Guo, W. Li, M. Qian, T. Jiang, P. Guo, Q. Du, N. Lin, X. Xie, Z. Wu, D. Lin, D. Liu, D-4F ameliorates contrast media–Induced oxidative injuries in endothelial cells via the AMPK/PKC pathway. Front. Pharmacol. 11, 556074 (2021). 33658920 10.3389/fphar.2020.556074 PMC7917283

[47] N. Ronda, F. Potì, A. Palmisano, R. Gatti, G. Orlandini, U. Maggiore, A. Cabassi, G. Regolisti, E. Fiaccadori, Effects of the radiocontrast agent iodixanol on endothelial cell morphology and function. Vascul. Pharmacol. 58, 39–47 (2013). 22985912 10.1016/j.vph.2012.08.005

[48] C.-F. Chang, X.-M. Liu, K. J. Peyton, W. Durante, Heme oxygenase-1 counteracts contrast media-induced endothelial cell dysfunction. Biochem. Pharmacol. 87, 303–311 (2014). 24239896 10.1016/j.bcp.2013.11.002 PMC3947226

[49] A. L. Ho, M. E. O’Malley, G. A. Tomlinson, Adverse events with universal use of Iodixanol for CT: Comparison with Iohexol. J. Comput. Assist. Tomogr. 31, 165–168 (2007). 17414747 10.1097/01.rct.0000237816.11054.09

[50] S. Sassa, Biological implications of heme metabolism. J. Clin. Biochem. Nutr. 38, 138–155 (2006).

[51] L. Ouyang, J. P. K. Armstrong, Q. Chen, Y. Lin, M. M. Stevens, Void-free 3D bioprinting for in situ endothelialization and microfluidic perfusion. Adv. Funct. Mater. 30, 1908349 (2020). 33071714 10.1002/adfm.201908349 PMC7116187

[52] D. B. Kolesky, K. A. Homan, M. A. Skylar-Scott, J. A. Lewis, Three-dimensional bioprinting of thick vascularized tissues. Proc. Natl. Acad. Sci. U.S.A. 113, 3179–3184 (2016). 26951646 10.1073/pnas.1521342113 PMC4812707

[53] L. Shao, Q. Gao, C. Xie, J. Fu, M. Xiang, Y. He, Directly coaxial 3D bioprinting of large-scale vascularized tissue constructs. Biofabrication 12, 035014 (2020). 32155602 10.1088/1758-5090/ab7e76

[54] I. S. Kinstlinger, G. A. Calderon, M. K. Royse, A. K. Means, B. Grigoryan, J. S. Miller, Perfusion and endothelialization of engineered tissues with patterned vascular networks. Nat. Protoc. 16, 3089–3113 (2021). 34031610 10.1038/s41596-021-00533-1

[55] C. Colosi, S. R. Shin, V. Manoharan, S. Massa, M. Costantini, A. Barbetta, M. R. Dokmeci, M. Dentini, A. Khademhosseini, Microfluidic bioprinting of heterogeneous 3D tissue constructs using low-viscosity bioink. Adv. Mater. 28, 677–684 (2016). 26606883 10.1002/adma.201503310 PMC4804470

[56] R. Levato, K. S. Lim, W. Li, A. U. Asua, L. B. Peña, M. Wang, M. Falandt, P. N. Bernal, D. Gawlitta, Y. S. Zhang, T. B. F. Woodfield, J. Malda, High-resolution lithographic biofabrication of hydrogels with complex microchannels from low-temperature-soluble gelatin bioresins. Mater. Today Bio. 12, 100162 (2021). 10.1016/j.mtbio.2021.100162 PMC8626672 34870141

[57] M. Wang, W. Li, J. Hao, A. Gonzales, Z. Zhao, R. S. Flores, X. Kuang, X. Mu, T. Ching, G. Tang, Z. Luo, C. E. Garciamendez-Mijares, J. K. Sahoo, M. F. Wells, G. Niu, P. Agrawal, A. Quiñones-Hinojosa, K. Eggan, Y. S. Zhang, Molecularly cleavable bioinks facilitate high-performance digital light processing-based bioprinting of functional volumetric soft tissues. Nat. Commun. 13, 3317 (2022). 35680907 10.1038/s41467-022-31002-2 PMC9184597

[58] C. Mandrycky, B. Hadland, Y. Zheng, 3D curvature-instructed endothelial flow response and tissue vascularization. Sci. Adv. 6, eabb3629 (2020). 32938662 10.1126/sciadv.abb3629 PMC7494348

[59] K. M. Chrobak, D. R. Potter, J. Tien, Formation of perfused, functional microvascular tubes in vitro. Microvasc. Res. 71, 185–196 (2006). 16600313 10.1016/j.mvr.2006.02.005

[60] B. D. Fairbanks, M. P. Schwartz, C. N. Bowman, K. S. Anseth, Photoinitiated polymerization of PEG-diacrylate with lithium phenyl-2,4,6-trimethylbenzoylphosphinate: Polymerization rate and cytocompatibility. Biomaterials 30, 6702–6707 (2009). 19783300 10.1016/j.biomaterials.2009.08.055 PMC2896013

[61] J. W. Nichol, S. T. Koshy, H. Bae, C. M. Hwang, S. Yamanlar, A. Khademhosseini, Cell-laden microengineered gelatin methacrylate hydrogels. Biomaterials 31, 5536–5544 (2010). 20417964 10.1016/j.biomaterials.2010.03.064 PMC2878615

[62] J. Baier Leach, K. A. Bivens, C. W. Patrick Jr., C. E. Schmidt, Photocrosslinked hyaluronic acid hydrogels: Natural, biodegradable tissue engineering scaffolds. Biotechnol. Bioeng. 82, 578–589 (2003). 12652481 10.1002/bit.10605

[63] D. Wangpraseurt, S. You, F. Azam, G. Jacucci, O. Gaidarenko, M. Hildebrand, M. Kühl, A. G. Smith, M. P. Davey, A. Smith, D. D. Deheyn, S. Chen, S. Vignolini, Bionic 3D printed corals. Nat. Commun. 11, 1748 (2020). 32273516 10.1038/s41467-020-15486-4 PMC7145811

[64] Q. Fang, Mesh-based Monte Carlo method using fast ray-tracing in Plücker coordinates. Biomed. Opt. Express 1, 165–175 (2010). 21170299 10.1364/BOE.1.000165 PMC3003331

[65] S. Jacques, T. Li, S. Prahl, mcxyz. c, a 3D Monte Carlo simulation of heterogeneous tissues (www.omlc.org/software/mc/mcxyz) (2013); omlc.org/software/mc/mcxyz.

[66] R. Poli, J. Kennedy, T. Blackwell, Particle swarm optimization. Swarm Intell. 1, 33–57 (2007).

[67] A. Jönsson, A. Toppi, M. Dufva, The FAST Pump, a low-cost, easy to fabricate, SLA-3D-printed peristaltic pump for multi-channel systems in any lab. HardwareX 8, e00115 (2020). 35498250 10.1016/j.ohx.2020.e00115 PMC9041223

