# Supplementary material for: High cell density and high-resolution 3D bioprinting for fabricating vascularized tissues
Source: Sci Adv. 2023 Feb 22;9(8):eade7923. doi: 10.1126/sciadv.ade7923 (PMC9946358; doi:10.1126/sciadv.ade7923)
Supplement: Supplementary file 1 — Figs. S1 to S10 Legends for data S1 to S3 [file sciadv.ade7923_sm.pdf]

Science Advances
AAAS

Supplementary Materials for

High cell density and high-resolution 3D bioprinting for fabricating vascularized tissues

Shangting You *et al.*

Corresponding author: Shaochen Chen, chen168@eng.ucsd.edu

Sci. Adv. **9**, eade7923 (2023)
DOI: 10.1126/sciadv.ade7923

The PDF file includes:

Figs. S1 to S10
Legends for data S1 to S3

Other Supplementary Material for this manuscript includes the following:

Data S1 to S3

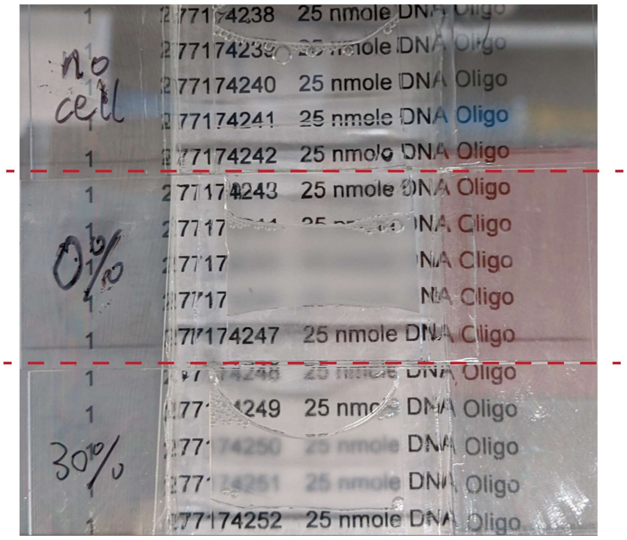

Fig. S1. Comparison of visual appearance among bioink with no cell (upper), with 40 million/mL cells (middle), and with 30% IDX and 40 million/mL cells (lower).

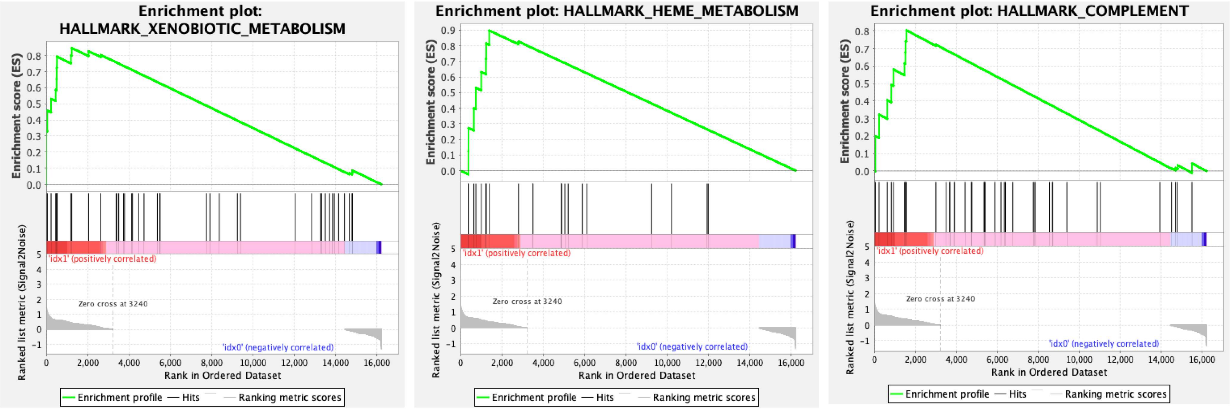

Fig. S2. GSEA enrichment plots.

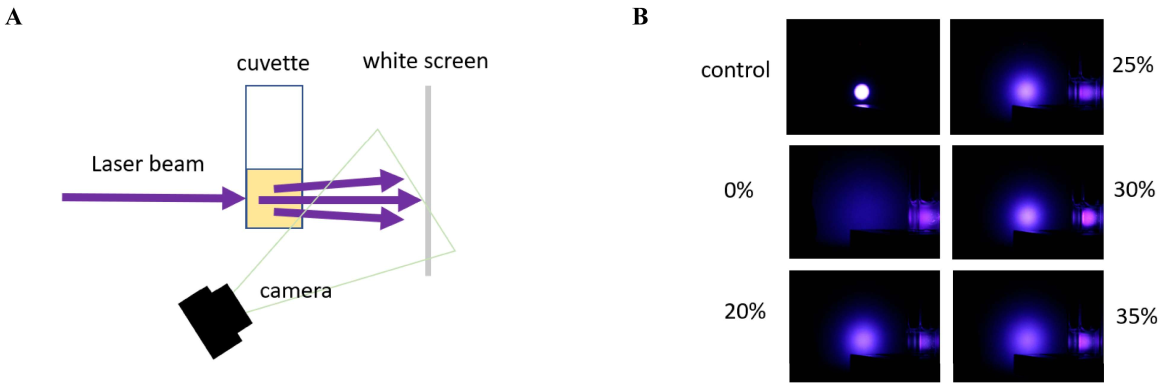

Fig. S3. A simple pilot experiment to determine the optimal IDX concentration. (A) Schematic of the setup. (B) Images captured by the exposure-locked camera.

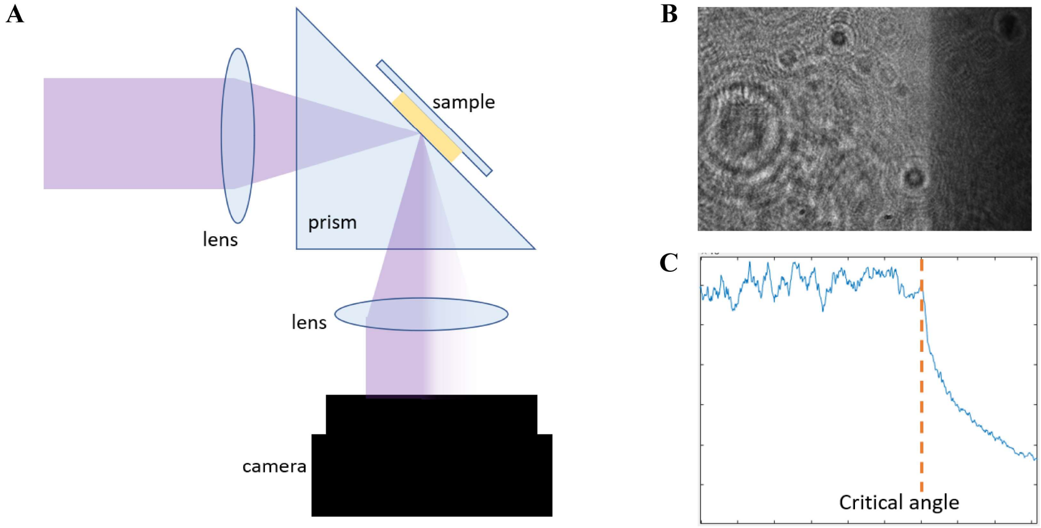

Fig. S4. Refractive index measurement. (A) A schematic showing the setup of the refractometer for refractive index measurement. (B) An example image captured by the camera. (C) Determining the critical angle of total internal refraction based on the image of (B).

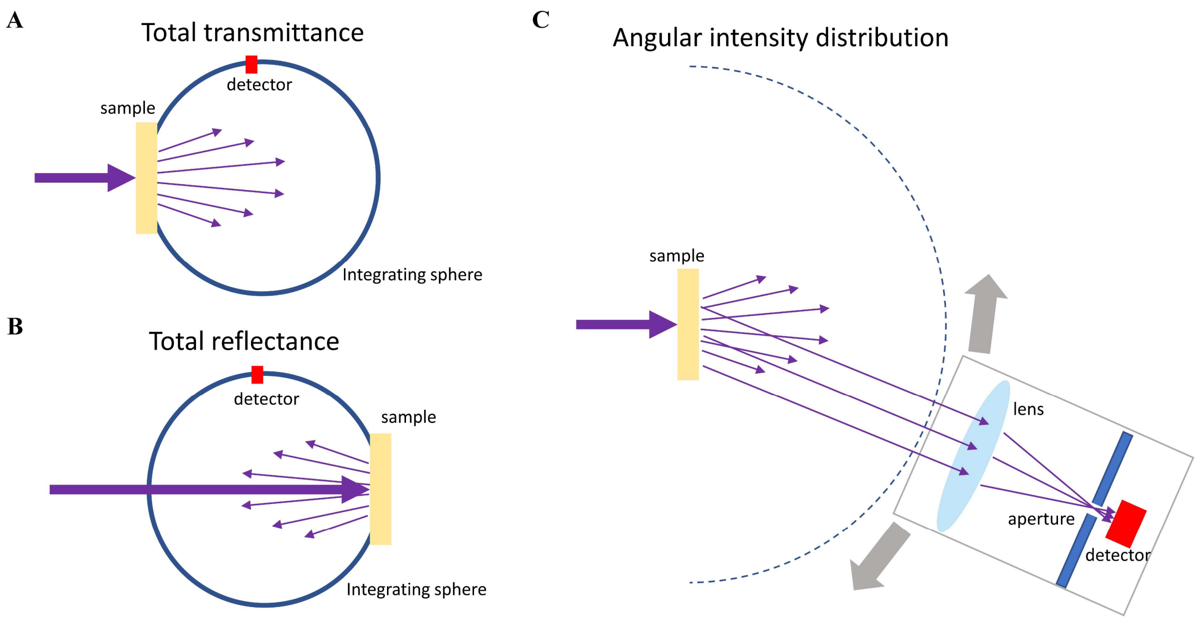

Fig. S5. Schematics showing measurement of optical properties of the cell-laden bioink samples. (A) Using an integrating sphere to measure the total transmittance. (B) Using an integrating sphere to measure the total reflectance. (C) Using an optical goniometer to measure the angular intensity distribution of the scattered light.

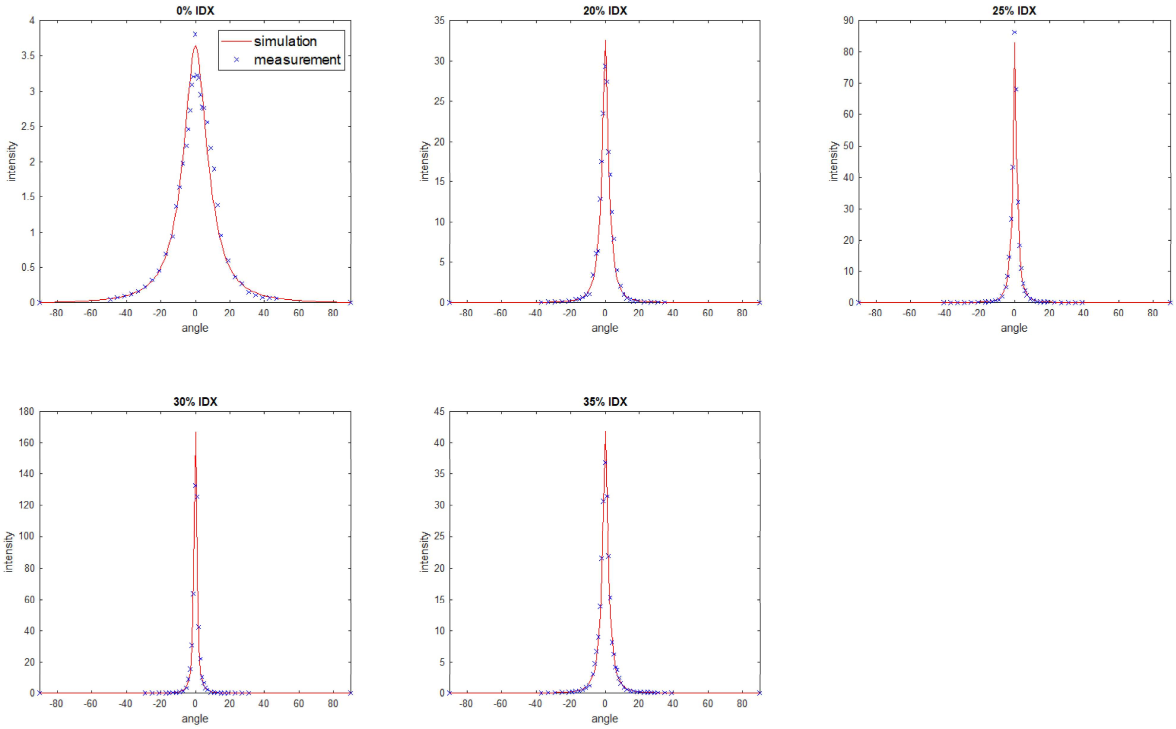

Fig. S6. Simulation data compared to the measured angular distribution of the scattered light.

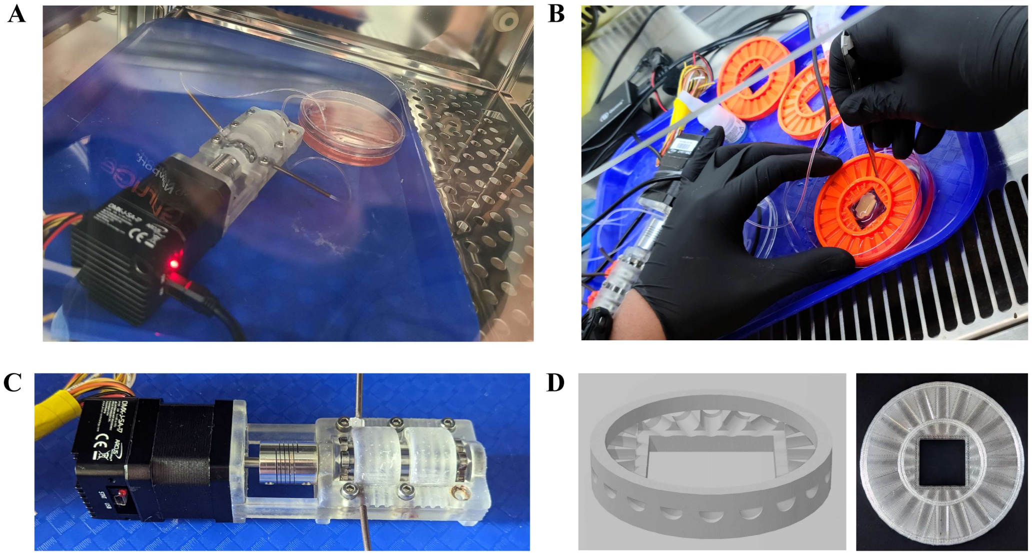

Fig. S7. Perfusion culture system setup. (A) The perfusion culture system in an incubator. (B) Setting up the perfusion culture system. (C) The multi-channel microfluidic peristaltic pump for the perfusion culture system. (D) The open-top fluidic manifold to anchor the samples and the tubings, avoiding relative motion between them.

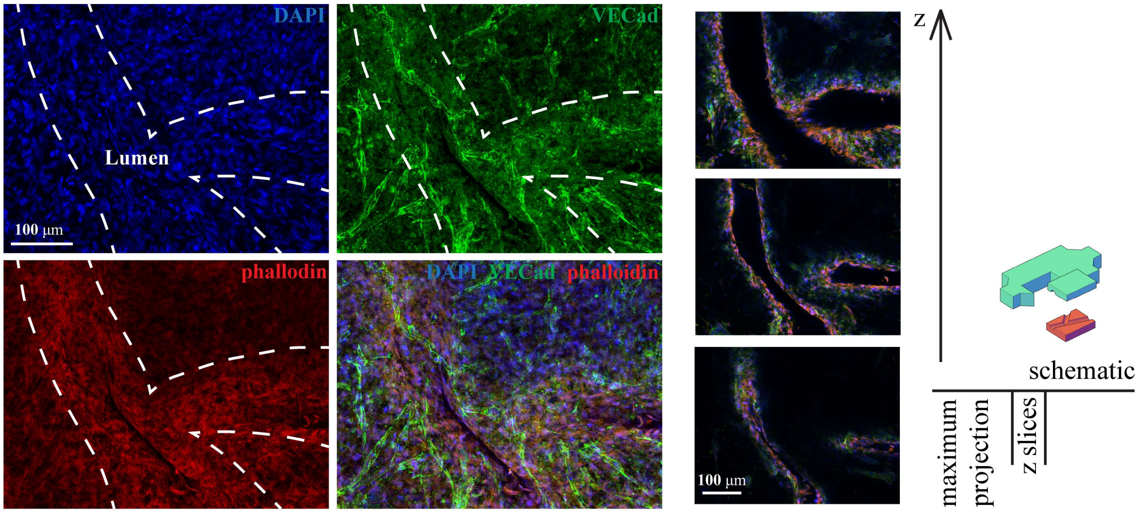

Fig. S8. Additional IF image of chunks showing endothelialization and angiogenesis.

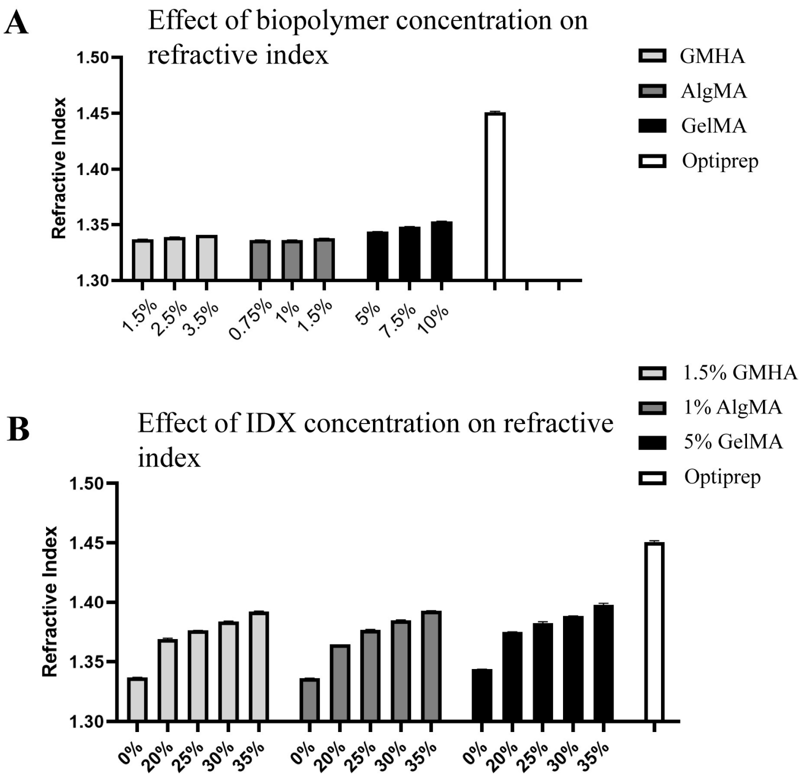

Fig. S9. Refractive indices of various bioink compositions. (A) Refractive indices of common bioinks at various biopolymer concentrations. (B) Refractive indices of common bioinks with various IDX concentrations.

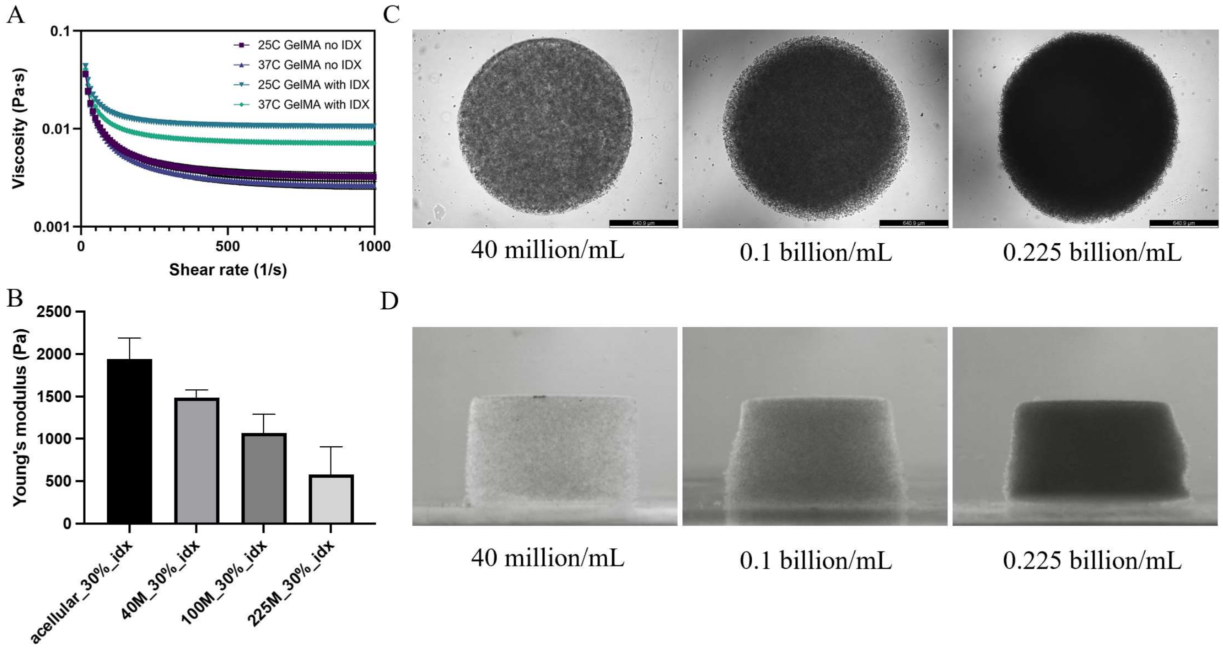

Fig. S10. Mechanical properties of the bioinks and printed structures. (A) Rheology properties of 5% GelMA bioinks with 30% IDX or without IDX, at 25°C or 37°C. (B) Young's moduli of the printed structure with 5% GelMA, 30% IDX, and various cell densities at 0, 40 million/mL, 0.1 billion/mL, and 0.225 billion/mL. (C) Top view image of the printed cylinder containing 40 million/mL, 0.1 billion/mL, and 0.225 billion/mL cells for Young's modulus testing. (D) Side view image of the printed cylinder containing 40 million/mL, 0.1 billion/mL, and 0.225 billion/mL cells for Young's modulus testing.

Data S1. (separate file) Significantly upregulated and downregulated genes.

Data S2. (separate file) Significantly upregulated and downregulated gene sets in the MsigDB curated database.

Data S3. (separate file) Significantly upregulated and downregulated gene sets in the MsigDB gene ontology database.
